# Novel Neuroprotective Multicomponent Therapy for Amyotrophic Lateral Sclerosis Designed by Networked Systems

**DOI:** 10.1371/journal.pone.0147626

**Published:** 2016-01-25

**Authors:** Mireia Herrando-Grabulosa, Roger Mulet, Albert Pujol, José Manuel Mas, Xavier Navarro, Patrick Aloy, Mireia Coma, Caty Casas

**Affiliations:** 1 Group of Neuroplasticity and Regeneration, Institut de Neurociències and Department of Cell Biology, Physiology and Immunology, *Universitat Autònoma de Barcelona* and *Centro de Investigación Biomédica en Red sobre Enfermedades Neurodegenerativas* (CIBERNED), Bellaterra, Barcelona, Spain; 2 Joint IRB-BSC-CRG Program in Computational Biology, Institute for Research in Biomedicine (IRB Barcelona), Barcelona, Catalonia, Spain; 3 Anaxomics Biotech SL, Barcelona, Catalonia, Spain; 4 Institució Catalana de Recerca i Estudis Avançats (ICREA), Barcelona, Catalonia, Spain; The University of Melbourne, AUSTRALIA

## Abstract

Amyotrophic Lateral Sclerosis is a fatal, progressive neurodegenerative disease characterized by loss of motor neuron function for which there is no effective treatment. One of the main difficulties in developing new therapies lies on the multiple events that contribute to motor neuron death in amyotrophic lateral sclerosis. Several pathological mechanisms have been identified as underlying events of the disease process, including excitotoxicity, mitochondrial dysfunction, oxidative stress, altered axonal transport, proteasome dysfunction, synaptic deficits, glial cell contribution, and disrupted clearance of misfolded proteins. Our approach in this study was based on a holistic vision of these mechanisms and the use of computational tools to identify polypharmacology for targeting multiple etiopathogenic pathways. By using a repositioning analysis based on systems biology approach (TPMS technology), we identified and validated the neuroprotective potential of two new drug combinations: Aliretinoin and Pranlukast, and Aliretinoin and Mefloquine. In addition, we estimated their molecular mechanisms of action *in silico* and validated some of these results in a well-established *in vitro* model of amyotrophic lateral sclerosis based on cultured spinal cord slices. The results verified that Aliretinoin and Pranlukast, and Aliretinoin and Mefloquine promote neuroprotection of motor neurons and reduce microgliosis.

## Introduction

Since Charcot first description, Amyotrophic Lateral Sclerosis (ALS) is considered an adult-onset disease characterized by severe degeneration of lower (spinal or bulbar) and upper (cortical) motoneurons (MNs). Approximately two thirds of ALS patients have a spinal form of the disease (limb onset) and present symptoms related to focal weakness and wasting which may start either distally or proximally in the upper and lower limbs. Paralysis is progressive and leads to death due to respiratory failure within 2–3 years for bulbar onset cases and within 3–5 years for limb onset [1]. Despite all the recent insights into the molecular mechanism of the disease, no improvement in the therapeutic options for ALS patients has been obtained during the past two decades. A large number of preclinical studies have been performed in rodent models of ALS to prevent, reverse or modulate the disease process. So far, none of these therapeutic strategies has been successfully replicated in clinical trials. As a consequence, besides riluzole, 2-amino-6-trifluoromethoxybenzothiazole [2], only palliative treatments are applied to improve the quality of life of ALS patients. Riluzole increases the life span of the patients by an average of 2–3 months [3], an effect that was reproduced in different clinical trials [4]. The mechanism of action (MoA) is linked to the blockade of sodium channels, inactivation of voltage-dependent calcium channels, and blockade of sodium-dependent glutamate release. The protective effect of riluzole has been examined in various models where excitotoxicity has an important role in MN death including organotypic-based models [5] and animal models of spinal cord injury [6].

In most cases (~90%), termed sporadic ALS (sALS), the etiology is unknown, although some inherited mutations found in familial cases (fALS) have allowed significant advances in the study of the etiopathogenesis during the last decades [7].

The discovery of new genes implicated in ALS and the existence of common mechanisms found in most of the genes have pointed to new insights into the pathogenic pathways. Bioinformatics analysis of their annotated functions reveal an enrichment in processes such as endocytosis and impaired vesicle (*Alsin*, vesicle-associated membrane protein-associated protein B/C (*VAPB)*, optineurin (*OPTN)*, transitional endoplasmic reticulum ATPase (*VCP)*), axonal transport and organelle trafficking (*spatacsin*), compromised autophagy (charged multivesicular body protein 2b *(CHMP2B*)) and protein degradation, in the unfolded protein response (ubiquilin-2 (*UBQLN2)*, sigma non-opioid intracellular receptor 1 (*SIGMAR1)*), transcription regulation and RNA metabolism (*C9orf72*, TAR DNA-binding protein 43 (*TARDBP)*, probable helicase senataxin (*SETX)*), as well as in mitochondria-dependent oxidative stress [8]. However, ALS is a heterogeneous disease, not only genetically, but also clinically, due to variability in several factors such as age, gender, site of onset and rate of progression. Hence, instead of targeting specific disease-causing genes an alternative is to target pathogenic mechanisms. Thus, the existence of several evidences for a complex interplay between glutamate excitotoxicity, mitochondrial dysfunction, glial activation, defective protein misfolding, oxidative damage and defective RNA processing highlights the value of molecular interaction profiles in the discovery of novel multicomponent therapies [9,10].

Systems biology has recently emerged as a new discipline that addresses this need by considering living organisms as networks of interacting genes, proteins and biochemical reactions. Given the complexity inherent to human physiology, systems biology tools are particularly suited for the identification of multi-targeted agents and drug synergistic effects. The complexity of ALS pathobiology prompted us to use computational biology and machine learning tools strategies to handle all data available and getting *in silico* relevant and integral information. Systems biology is making important contributions to biomedical research [11], and it may play a pivotal role in the future of drug discovery [12–14]. Recent drug development strategies against Charcot-Marie-Tooth disease type I (CMT1A) and breast cancer highlight the power of network pharmacological approaches for identifying drug combinations with high clinical efficacy and low side effect risks [13]. Systems biology approaches have also been successfully applied to identify novel proteins involved on Alzheimer’s disease [15,16]. Herein, we have analysed public available data through systems biology approach to identify novel combinations of repositioned drugs with neuroprotective potential for ALS.

## Materials and Methods

### Systems Biology analysis for multicomponent drug discovery

TPMS (Anaxomics Biotech, Barcelona, Catalonia, Spain) is a top-down systems biology approach with potential applications in drug repositioning [12,17,18].

#### Characterization of ALS

Through manual curation of the literature, we defined a set of restrictions characterising ALS, which were used to build the protein network and the mathematical model around ALS. We identified the main pathophysiological processes described to be involved in ALS: glutamate excitotoxicity, protein misfolding and aggregation, mitochondrial dysfunction, oxidative stress, defective RNA processing and glia activation. Subsequently, each pathophysiological process was further characterized at protein level. A total of 72 proteins were used to focus the analysis on ALS in the human biological network (Table 1). The details about the Uniprot ID of each protein and the bibliographic reference linking them with ALS can be found in **S1 Table**.

**Table 1:** Human biological network focused on ALS.

ALS pathophysiological motives	N° of seeds	Gene name
Glutamate excitotoxicity	11	**SLC1A2, GRIA1, GRIA2, GRIA3, GRIA4, SOD1, GRIN1, GRIN2A, GRIN2B, GRIN2C, GRIN2D**
Protein misfolding and aggregation	23	**SOD1, RNF19A, DERL1, MAP3K5, TARDBP, VCP, CSE1L, GRN, KPNA2, FUS, TNPO1, OPTN, NEFH, PRPH, FIG4, BICD2, ALS2, MTOR, SQSTM1, VAPB, CHMP2B, HSPA1A, HSPA1B**
Mitochondrial dysfunction	17	**SOD1, KIF5B, MAPK11, MAPK11, MAPK12, MAPK13, MAPK14, DCTN1, KIFAP3, BCL2, Bcl-xL, XIAP, BAD, BAX, cytochrome c, caspase-3, caspase-9, ANG**
Oxidative Stress	6	**SOD1, RAC1, NOX1, ERO1L, P4HB, PDIA3**
Defective RNA processing	9	**ANG, ELP3, FUS, FUS, SETX, SMN1, TARDBP, RGNEF, ATXN2, C9orf72**
Glial activation	15	**SLC1A2, SOD1, VEGFA, VEGFA, VEGFB, TNF, IL1B, CHGA, CHGB, SCG2, CD14, TLR2, IL4, IGF1, CX3CR1, CX3CL1**
**Total number of seeds**	**81**	
**Unique seeds**	**72**	

The human biological network incorporates all the available relationships between proteins from a regularly updated in-house database drawn from public sources: KEGG [19], REACTOME [20], INTACT [21] BIOGRID [22], HDPR [23], MATRIXDB [24], MIPS [25], DIP [26], MINT [27]. At the same time, the network was embedded with all sorts of biological information (drug targets, tissue expression, biomarkers) about nodes (i.e. proteins) and edges (i.e. connections) from public sources. The analysis of the network was focused in the area around the proteins identified as important in ALS pathophysiology, including 2,455 proteins and 65,044 relationships (30.228 from KEGG_XML, 6.651 from HPRD, 1.030 from MatrixDB, 21.572 from Reactome, 8.908 from BIOGRID and 11.711 from INTACT). Average links per node were 30,6.

**Fig 1:**
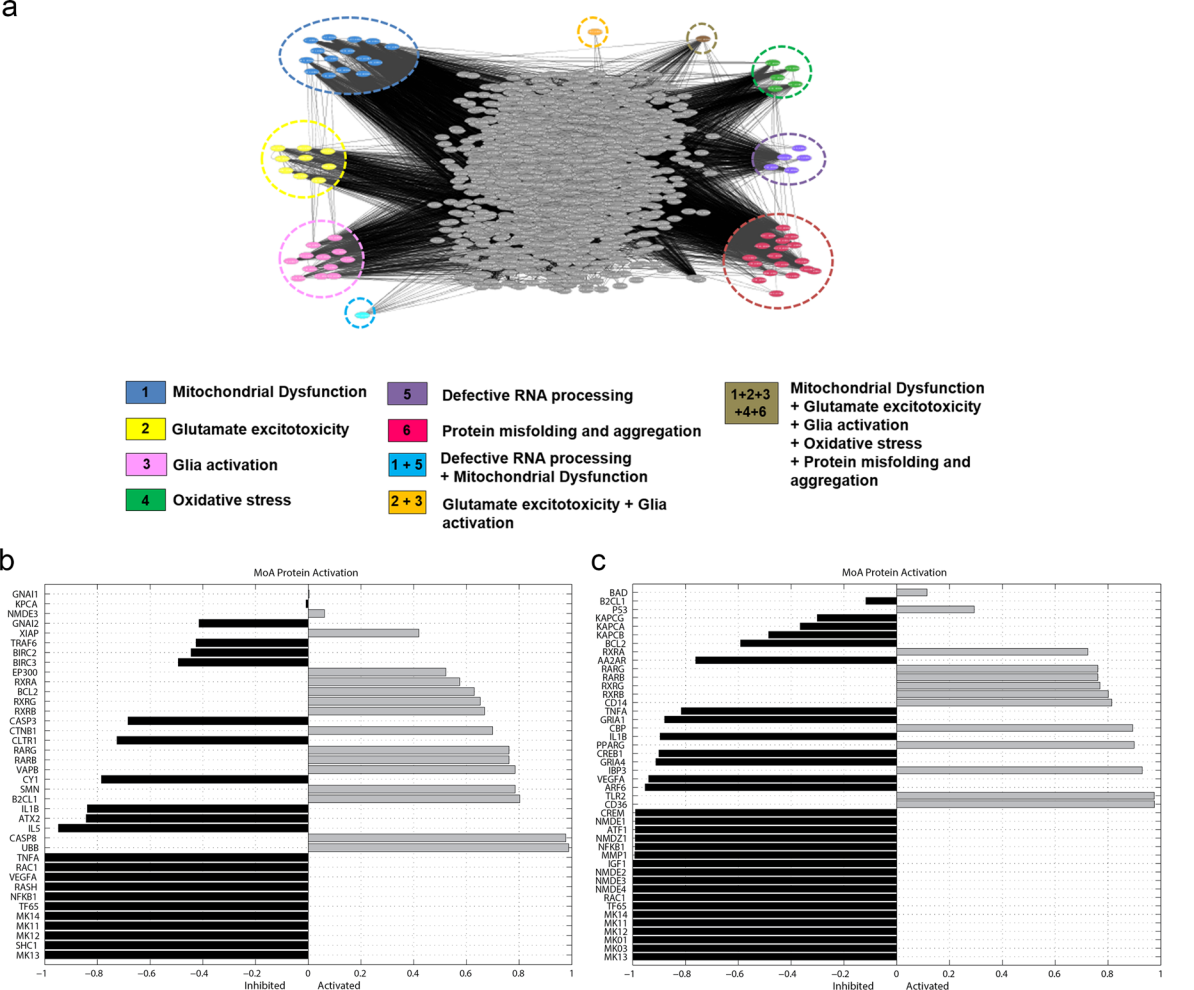
Predicted MoA of CD1 and CD2 multicomponent drugs. **a.** Snapshot of ALS network: snapshot of the full network visualized through the cytoscape software platform. Nodes (traingles) represent proteins and links are functional interactions between proteins. The effector proteins of the different pathophysiological motives (or effectors shared by several motives) are grouped and labelled in different colors. **b and c.** The graphs show the level of activation or inhibition of each node depicted in the MoA representation of CD1 (**b**) and CD2 (**c**). The bars in grey correspond to proteins that are calculated to be activated after the stimulation, and the bars in black correspond to proteins calculated to have reduced activity after the treatment with the stimulus. The activity status of the proteins is the average of all the individuals included in the analysed population, and it is represented between 1 and -1, 1 meaning activation and -1 inhibition of the gene/protein activity respect to the rest of the proteins involved in the MoA.

The complexity of the network generated can be perceived in Fig 1A where a snapshots of the full network visualized through the cytoscape software platform [28] is shown. For visualizing, modeling and analyzing molecular interaction of ALS network, the.cys version of the full network is provided as Supp. Material. VCP, TARDBP, RGNEF are not included in the cytoscape ALS interactome since they are highly connected proteins (more than 200 known relationships), and FUS since there are not available information in public databases.

#### Repositioning analysis

Two complementary strategies are used to generate mathematical models of living systems: an Artificial Neural Network (ANN) and Sampling Methods. ANNs are supervised algorithms which identify relations between drug targets and clinical elements of the network that are used for training a classifier with the information contained in DrugBank about drugs and indications [29,30], with capacity for predicting and scoring novel potential drugs. The accuracy of the ANNs to reproduce the indications of Drugbank is 98% for those drugs with all targets in the human biological network after applying a cross-fold validation process.

A total of 5440 drugs that generated approximately 14,794,080 of binary combinations were screened, and some pre-analysis filters were applied (**S5 Table**). Combinations composed by drugs with an outstanding safety profile with at least one known human target, with known interactions and the known therapeutic target are inside the human biological network were analysed. Within these 5440 drugs, there are subsets of drugs that share the target profile; these drugs are considered as DrugBank synonyms. The drugs that share the target profile with another one are not considered in the analysis, but are provided for the final drug candidate selection. A total of 134.940 binary combinations composed by non-synonymous drugs were screened.

Combinations composed by approved drugs were sorted and selected by its relationship with ALS or its corresponding motive, high synergism and their ability to cross the blood brain barrier. Drugs with poor safety profile were further filtered out and those combinations previously related to ALS were also excluded. A total of 12 combinations Pimecrolimus + Pranlukast; Alitretinoin + Pranlukast; Alitretinoin + Acamprosate; Thiethylperazine + Alitretinoin; Raloxifene + Chloroquine; Pimecrolimus + Chloroquine; Pimecrolimus + Rasagiline; Acamprosate + Cinnarizine; Lisuride + Acamprosate; Acamprosate + Carphenazine; Methylergonovine + Acamprosate; Mefloquine + Alitretinoin. Lastly, we selected two combinations of repositioned drugs: Alitretinoin plus Pranlukast (CD1) and Alitretinoin plus Mefloquine (CD2) that fulfilled all the criteria and had a novel, potentially beneficial MoA against ALS.

Sampling methods were used to describe with high capability all plausible relationship between sets of proteins previously identified with ANNs. Sampling Methods generate mathematical models that comply with a given set of restrictions, corresponding to the available biological knowledge about the constructed networks, together with knowledge derived from DrugBank and GEO [31]. As the number of restrictions is always smaller than the number of parameters required by the algorithm, any process modelled by TPMS has a “population” of different solutions, which is set around 10^6^–10^9^, since this interval is estimated to faithfully portray nature. Consequently, models result in both “global” predicted MoA, which account for the majority of the population, and “cluster” mechanisms of action, which are more accurate for population subgroups. A normalized synergism score (SE) is provided for the protein involved in the synergism between the two drugs of the combination. SE accounts for the ponderation of the number of solutions that present the node of interest being affected by both drugs, either in an additive, synergistic or antagonistic way, and the synergistic effect over the node of both drugs (rather than additive). The maximum score according to this calculation is 0.5.

The MoA is validated in a two-step process. First, we checked that each link was accurate, i.e., was already described in the literature. Second, we checked that the MoA made sense overall, featuring pathways coherent with the living system, the combinations of repositioned drugs used for treatment and the known pathophysiology of ALS.

### Spinal cord organotypic cultures

We used organotypic-based spinal cord cultures prepared from lumbar spinal cords of 8-day-old Sprague-Dawley rat pups as previously described [32–34]. Briefly, lumbar spinal cords were collected from pups and placed in ice-cold high glucose-containing (6.4 mg/ml) Gey’s Balanced Salt Solution (GBSS) (Sigma-Aldrich, Steinheim, Germany). After removing meninges and roots we cut the spinal cords into 350 μm transverse sections with a McIlwain Tissue Chopper. We transferred four sections onto 30-mm-diameter Millipore Millicell-CM culture (0.4 μm, Millipore, Billerica, USA) placed into 6-well plates (Thermo Fisher Scientific, Waltham, MA, USA) containing 1 ml of culture medium, consisting in 50% (v/v) minimal essential medium (MEM), 25 mM Hepes, 25% (v/v) heat-inactivated horse serum, 2 mM glutamine and 25% (v/v) Hank’s Balanced Salt Solution (HBSS, Sigma) supplemented with 25.6 mg/ml glucose; pH 7.2. Cultures were maintained at 37°C in a 5% CO_2_/95% air humidified environment. We left cultures to stabilize for 1 week, and then changed the medium twice per week. Excitotoxic insult consisted in the addition of 100 μM threohydroxyaspartate (THA) (for 1 to 4 weeks) [33]. We added concomitantly single drugs or in combination to assess the neuroprotective effect *vs* their vehicle controls (**S2 Table**); addition of riluzole (5 μM) was also assayed as positive control. Five cultures were performed in all experiments. The procedures involving animals were approved by the Ethics Committee of *Universitat Autònoma de Barcelona* and followed the European Communities Council Directive 2010/63/EU.

### Immunohistochemistry

We fixed slices maintained in the different experimental conditions with 4% paraformaldehyde in phosphate-buffered saline for 1h at RT. After blocking with 5% normal donkey serum (Vector Laboratories, Burlingame, CA, USA) and 0.2% Triton-X-100 in PBS (PBS-TX), we incubated the sections with primary antibodies against anti-neurofilament heavy chain (NF-H or SMI-32, 1:8000) and rabbit anti-ionized calcium binding adapter molecule 1 (Iba-1, 1:200) (Wako, Tokyo, Osaka, Japan). For immunofluorescence, we thoroughly washed the cultures in PBS with 0.2% Tween-20 (PBS-T) and incubated them with appropriate secondary antibody Alexa Fluor^®488^ donkey anti-rabbit IgG (1:500) and Alexa Fluor^®594^ donkey anti-mouse IgG (1:500) (Invitrogen Corp.; Carlsbad, CA, USA), diluted in PBS-T for 1 h at RT. Finally, cell nuclei were labelled with DAPI (1:2000) for 1 min in PBS and the sections mounted with Fluoroamount-G medium (SouthernBiotech, Birmingham, AL, USA). We analysed the slides under confocal microscope (Confocal Laser Scanning Microscope Zeiss LSM 700; Zeiss, Jena, Germany).

### Motoneuron counting and image analysis

We identified MNs in the slices by immunostaining with SMI-32 antibody and on the basis of their morphology and size (Ø 25 μm) and their localization in the ventral horn of lumbar sections. All thoracic and sacral sections were out of counting. We blindly counted MNs meeting these criteria in each spinal cord section. To count SMI-32 positive cells in the ventral horn of organotypic spinal cord slices we used Z-stack confocal series. To quantify Iba-1 immunoreactivity we selected the ventral horn of each spinal cord section. The microphotographs were transformed to a grey scale and analyzed using ImageJ software. Immunoreactivity was assessed by calculating the integrated density, after defining a threshold for background correction.

### Statistical analysis

Data are shown as mean ± SEM. An average of n = 5-days experiment were performed with n = 4–5 biological replicates each day. Statistical significance (p<0.05) between culture treatments was determined by one-way ANOVA followed by Dunnett’s post-hoc test.

## Results

### Identification of putative neuroprotective polypharmacology for ALS

The repositioning analysis based on systems biology approach applying ANN identified two drug combinations from the repository: Alitretinoin plus Pranlukast (CD1) and Alitretinoin plus Mefloquine (CD2). They may be potentially useful to treat ALS, with a focus on tackling the disease from several angles at a time.

Aliretinoin is an active agent to treat psoriasis with an agonistic effect on retinoic receptors that could activate spinal cord repair mechanisms and modulate the inflammatory reaction [35–37]. Because of its potential benefits on neuroprotection, alitretinoin was included in both combinations CD1 and CD2. In CD1 included also Pranlukast, an agent used against ashma and allergy that exerts anti-inflammatory effects because it inhibits CysLT1 receptor, nuclear factor kappa B (NFƙB) and TNFα, suggesting a possible suppressive effect on the spinal cord inflammation and neutrophil infiltration [38]. CD2 includes Alitretinoin combined with Mefloquine, employed to treat malaria, which may prevent glia activation and glutamate excitotoxicity.

### Drug combinations exert motor neuron protection

**Fig 2:**
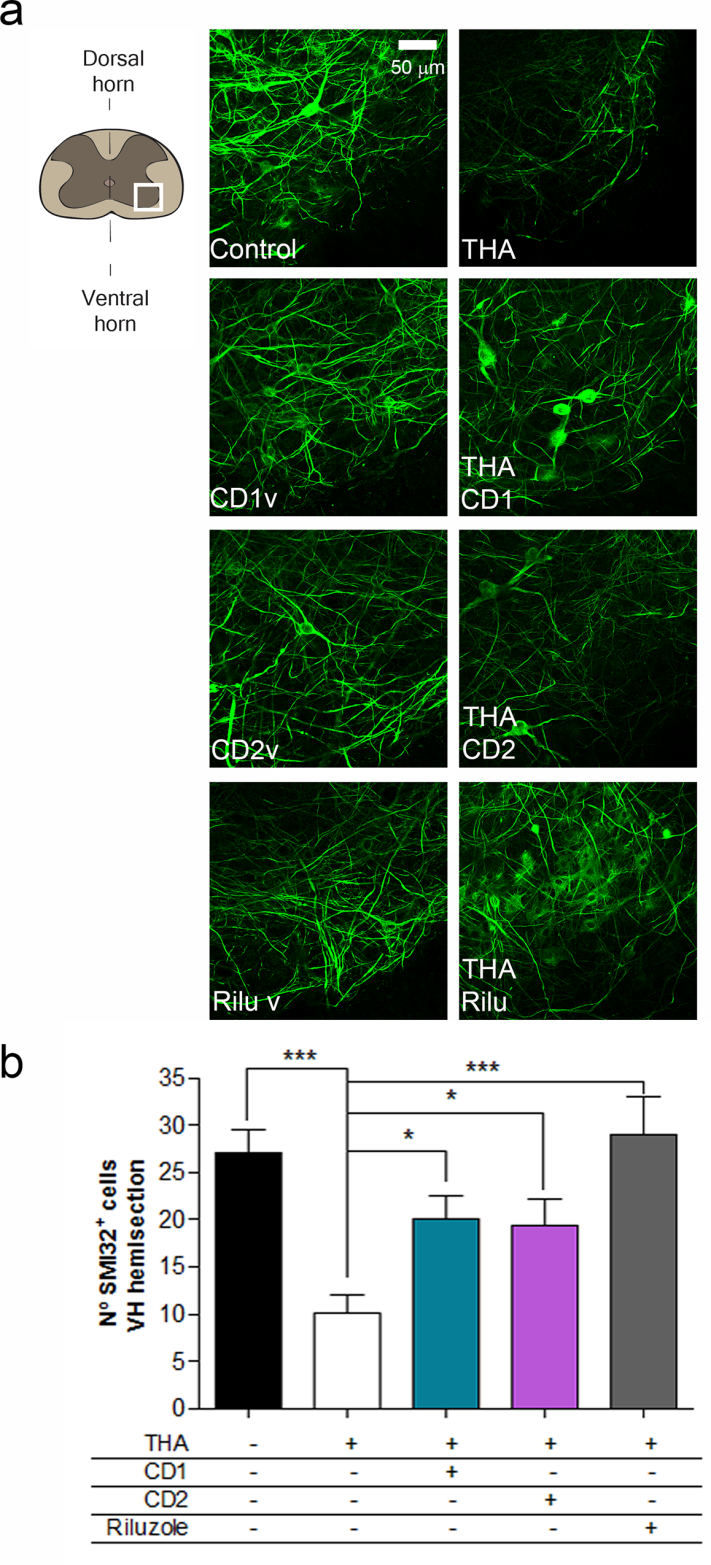
*In silico* design of polypharmacology for neuroprotection in an *in vitro* model of ALS. **a.** *Left*, Schematic drawing indicating the site of analysis (white frame) of MN survival at the ventral horn of the spinal cord slice. *Middle and right*, Representative microphotographs of MNs in the ventral horn of the spinal cord slice detected by immunohistochemistry with the SMI-32 antibody at 4 weeks after THA treatment. *Mid panels* show control culture and with addition of vehicle (v) for each drug combination (CD1-CD2) or riluzole. Right panels show cultures subjected to excitotoxicity by THA alone or with co-treatment with CD1 and CD2 drug combinations or riluzole. Scale bar, 50 μm. **b.** Bar graph showing the number (mean±SEM, n = 5) of SMI-32 positive cells in the ventral horn of each spinal cord slice. (***p<0.001; *p<0.05 by Dunnett’s post-hoc test vs THA condition).

In order to perform a preclinical screening of the selected drug combinations we used an spinal cord organotypic-based culture (SCOCs) because it retains many organizational features of the spinal cord, such as neuronal circuitry, relatively well-preserved cellular architecture and glial–neuronal interactions [5,32,34]. A well-established in vitro model for ALS is obtained by producing progressive chronic glutamate excitotoxicity by the addition of THA, an inhibitor of glutamate transport, in the culture [33,39–42]. After 7DIV, we administered THA accompanied by either vehicle or each drug combination at estimated optimal concentrations. We estimated these concentrations by inferring the information available from clinical uses and pharmacokinetic studies in rat. After 4 weeks post-excitotoxic insult controls and vehicle-treated SCOCs showed similar numbers of surviving MNs (Fig 2A). As expected, we observed a significant reduction in the number of MNs in the slices treated with THA alone (10±2) compared with control slices (27±2). In contrast, slices treated with THA plus CD1, CD2 or riluzole (5 μM), used as a positive control, presented a significant neuroprotective effect on MNs preservation (20±2, 19±3 and 29±4, respectively) (Fig 2A–2B).

**Fig 3:**
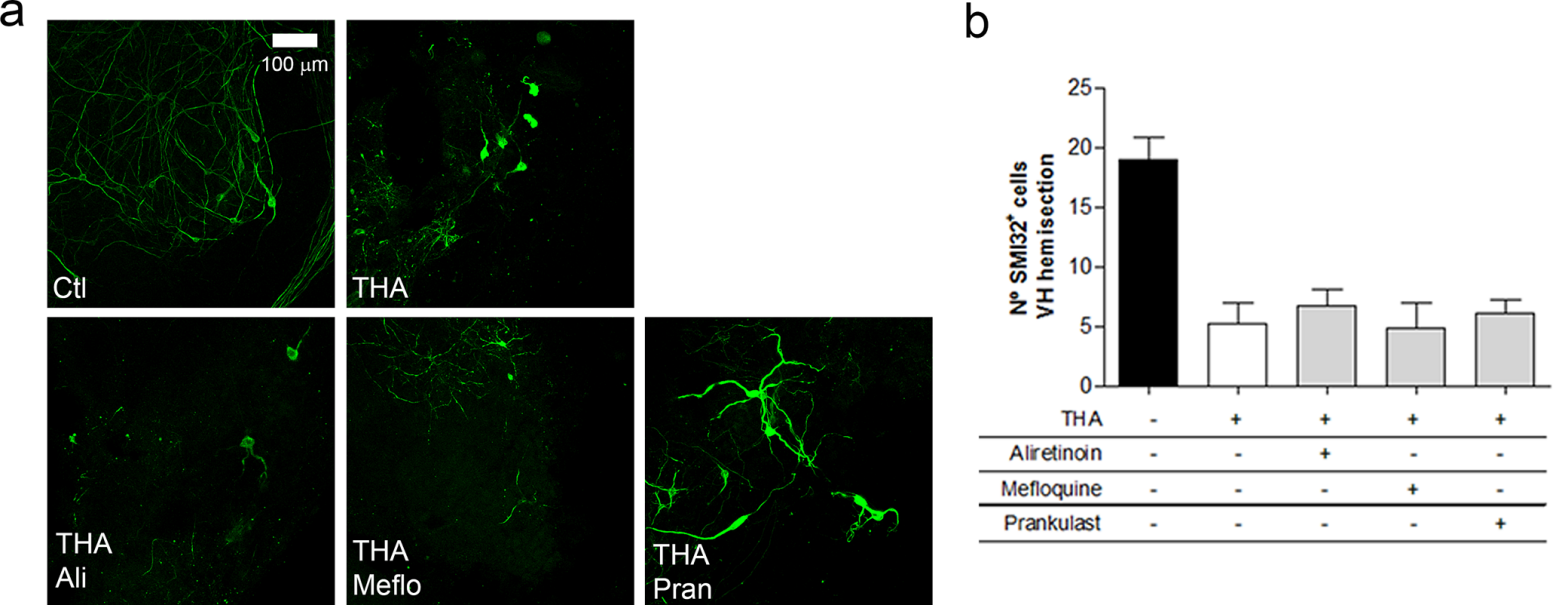
Lack of neuroprotection exerted by single drugs. **a.** Representative microphotograhs of SMI-32 at 4 weeks after excitotoxic treatment stained MNs after co-treatment with THA plus CD1 and CD2 components: Aliretinoin (Ali), Mefloquine (Meflo) and Pranlukast (Pran). **b**. Bar graph showing the number of SMI-32 positive neurons in the ventral horn of each spinal cord slice obtained after each treatment. (mean±SEM, n = 5). No significant differences comparing each drug treatment by Dunnett’s post-hoc test vs THA condition.

To test the synergism of the combined compounds, we analysed the effects on MNs survival on treated-SCOCs with each separate drug of CD1 and CD2 combinations: Mefloquine, Aliretinoin and Pranlukast. We found that, while combinations preserve MNs, each compound alone had no neuroprotective effect on the excitotoxic-injured SCOCs (Fig 3). MNs treated with single drugs showed disrupted neurites compared with controls and they presented a degenerating appearance like control THA-treated slices.

### Molecular mechanism prediction

Sampling methods were applied to identify the mechanisms of action of the three drugs selected from the reprofiling results. 181 proteins have been identified in the MoA of CD1, corresponding to the synergic action of pranlukast and alitretinoin (**S3 Table**). 94 proteins have been identified in the MoA of CD2, corresponding to the synergic action of mefloquine and alitretinoin (**S4 Table**). We observed that both drug combinations downregulated all forms of p38MAPKs proteins, called as MK 11–14, which have been already reported to have a role in ALS pathology and in inflammation (Fig 1B and 1C) [43–45].

### Drug combinations reduce microglial reactivity

**Fig 4:**
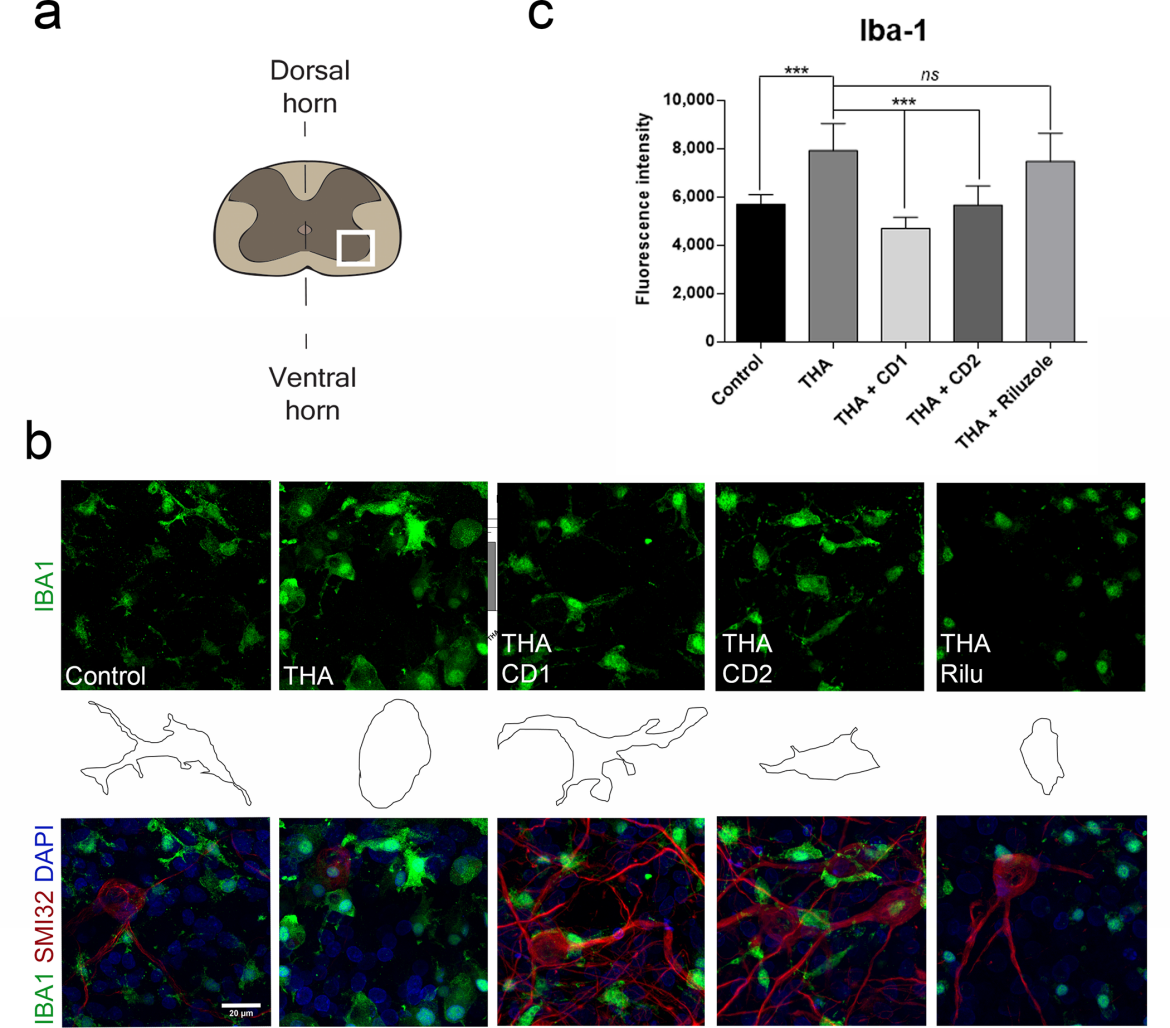
Reduction of microgliosis by drug combinations CD1 and CD2. ***a*.** Schematic drawing indicating the site of analysis (white frame) of microgliosis at the ventral horn of the spinal cord slice. **b**. *Top panels* are representative microphotographs showing microglia stained with anti-Iba1 (green) at 3 weeks post-THA treatment alone or co-treated with each drug combination or riluzole. *Middle panels* are profile drawings of microglia showing the ameboid-like or ramified shape acquired after each treatment. *Bottom panels*, representative microphotographs of the merge fluorescent staining with anti-Iba1, DAPI and MNs stained with SMI-32. Scale bar, 20 μm. **c.** Bar graph showing the microglial reactivity of each experimental condition by measuring the immunofluorescence intensity of Iba-1 in the ventral horn of each spinal cord slice. (mean±SEM, n = 5) (***p<0.001; by Dunnett’s post-hoc test vs THA condition).

**Fig 5:**
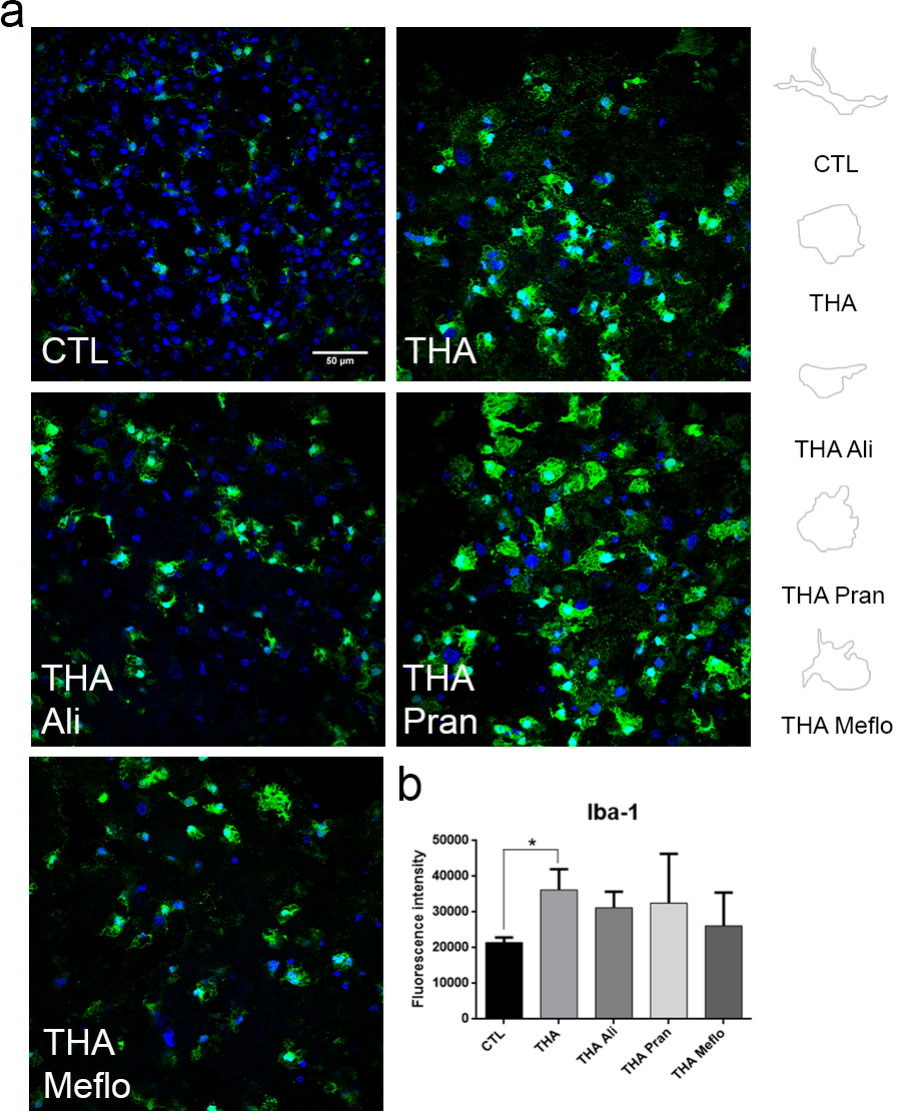
Lack of anti-inflammatory effect exerted by single drugs. **a.** Representative microphotograhs of microglia and DAPI at 3 weeks after co-treatment with THA alone or plus single components of the neuroprotective combinations (CD1 and CD2): Aliretinoin (Ali), Mefloquine (Meflo) and Pranlukast (Pran) (n = 5). *Right panels* are profile drawings of microglia showing the ameboid-like or ramified shape acquired after each treatment. **b.** Bar graph showing the microglial reactivity of each experimental condition by measuring the immunofluorescence intensity of Iba-1 in the ventral horn of each spinal cord slice. (mean±SEM, n = 5) (*p<0.05; by Dunnett’s post-hoc test vs THA condition).

Since glial cells are important contributors to inflammatory state and ALS pathology we analysed microglial reactivity and morphology in SCOCs by immunohistochemistry (Fig 4). Slices treated with the different vehicles presented the same pattern of Iba-1 labeling, and microglial morphology that control cultures (not shown), corresponding to resting microglial cells with small cell bodies and thin processes. Reactive microglia increase in size and thicken and shorten their processes in early stages to become amoeboid microglia with phagocytic activity [46,47]. We observed that microglia became reactive with amoeboid morphology due to THA treatment of the slices (Fig 4). The slices treated with THA plus CD1 or CD2 presented a phenotype ramified-resting and similar to control SCOCs (Fig 4B, middle panel). Addition of the single compounds, Mefloquine, Aliretinoin and Pranlukast to the injured cultures did not prevent the amoeboid reactive microglia phenotype induced by THA treatment (Fig 5). These results suggest that CD1 and CD2 are promising for reducing microgliosis in ALS, but not the individual components.

## Discussion

Systems biology was used as strategy to identify new combinatorial drugs with enough evidences to be promising for the treatment of human patients with ALS. In addition, we described their MoA and point to new human signalling targets for ALS. The up-to-date technology and filters used may allow for quickly move towards phase II/III trials in their way to clinical translation of the drug combinations composed by Alitretinoin plus Pranlukast (CD1) or Alitretinoin plus Mefloquine (CD2). However, further experiments *in vivo* should be done in to perform a safe translation to human.

Failure of multiple treatment options proposed for ALS to have a positive outcome in clinical trials has led to a frustrating reduction of trials. Many treatment assays have been based on the knowledge of etiopathogenic events mostly found by using transgenic mice carrying superoxide dismutase 1 (SOD1) ALS linked mutations. Moreover, lack of reliable early biomarkers makes it difficult early treatment, which it is often initiated in late phases of the disease. Our study is based on data gathered from human pathology and models in their late stages, thus searching for potential treatments that may be useful in the clinical setting.

Our blind screen of existing compounds against the multitude of ALS-specific targets identified possible combinations of off-patent drugs in cell-based assays. Previous similar studies had found that two drugs with different indications (an antipsychotic and an antiprotozoal) exhibit an unexpected anti-tumoral activity [48]. We found that two predicted combinations target two of the main processes related to ALS: excitotoxicity and neuroinflammation. Related to the former, as mentioned, riluzole, the only compound that presents limited benefits in ALS patients, exerts it effect mainly by reducing excitotoxicity. Other compounds such as ceftriaxone that specifically increases the expression of glial glutamate transporters in astrocytes, prolongs survival in the tgSOD^G93A^ [49]. However, although phase I and II clinical trials were successful, a phase III clinical trial investigating the effect of ceftriaxone was negative [50]. On the other hand, microglia actively contributes to the disease pathogenesis, since specific deletion of mutant SOD1 in microglia significantly prolongs survival in the tgSOD^G93A^ model [51]. Microglia acquires an activated state during the disease process [52], characterized by cell body enlargement and thick processes, and get properties of antigen-presenting cells and start to interact with T-cells, which infiltrate the affected spinal cord and cortex [53,54]. This microglial activation starts shortly before clinical onset of the disease, and the number of activated glia and infiltrated T-cells increases with disease progression [53,55]. Different strategies targeting inflammation have already been investigated. General immunosuppression or immunomodulation has been ineffective [56]. Celecoxib, a cyclooxygenase 2 (COX2) inhibitor, was effective in the tgSOD^G93A^, but not in patients [50]. Attempts to neutralize the released ROS with vitamin E, creatine or coenzyme Q10 (CoQ10) were unsuccessful in patients [4].

TPMS technology is capable to reveal drugable signalling targets and screen drug libraries to end up with effective combinations for complex pathological situation. We found that the combined use of Alitretinoin and Pranlukast (CD1) produced MN preservation and reduced microgliosis in the ALS in vitro model. Pranlukast is a selective antagonists of the cysLTR1 and can modulate inflammation though cyclic nucleotide phosphodiesterases, 5’-lypoxigenase and by suppressing the proinflammatory transcription factor NFƙB [57,58]. Alitretinoin or 9-cis-retinoic acid is a panagonist retinoid, capable of binding to all six known retinoid receptors. Aliretinoin is used to improve skin-related diseases such as dermatitis or Kaposi’s sarcoma [35]. However, neither of these drugs exert neuroprotection when administered alone. Curiously, the benefits obtained by Alitretinoin were also obtained with the simultaneous addition of Mefloquine, a 4-quinolinemethanol antimalarial and antiparasitic drug structurally related to quinine.

Both drug combinations tested prevented MN death induced by chronic excitotoxicity. Such effect may be associated to anti-excitotoxic effect by inhibiting p38MAPK. Rho-mediated calcium-dependent activation of p38αMAPK has been described as a trigger of excitotoxic cell death [43]. The MoA analyses indicate that both drug combinations exert an inhibitory effect over the four p38MAPK proteins: β, γ, α and δ (on results named as MK11, MK12, MK13 and MK14). It is known that persistent activation of p38MAPK in the SOD1^G93A^ mice correlates with disease progression [44], and that its inhibition in an in vitro excitotoxicity ALS model rescues MNs from cell death [45].

Regarding the inflammatory response, both combinations may regulate pro-inflammatory cytokines, such as interleukine-1β (IL1-β) and TNF-α. At late stages of ALS, microglia become non-responsive and dystrophic, a phenotype that follows the chronic activation and the release of these pro-inflammatory cytokines. Activation of TNFR1 in neurons classically leads to apoptosis but also increases AMPA receptors in the membrane [10]. We observed that combinations CD1 and CD2 promoted a shift to a resting microglia phenotype concomitant with a slight reduction of TNFα production which will be likely beneficial in helping MN survival. Microglia present an extremely cellular plasticity switching from a “resting” phenotype with fine processes to a globular “amoeboid” activated phenotype with the ability to migrate to sites of damage, phagocyte cellular debris and have toxic effects on neurons.

The dual treatment with Aliretinoin and Mefloquine (CD2) may induce the activation of the heterodimer composed by peroxisome proliferator-activated receptors (PPARG), retinoid X receptor (RXR) and transmembrane phosphoprotein (CBP). PPARs are ligand-activated transcription factors that play pivotal roles in the regulation of a large number of biological processes including inflammation. PPARs readily heterodimerize with the RXR prior to ligand binding [59]. After ligand binding, the PPAR/RXR heterodimer stably binds on genomic DNA at specific sites called peroxisome proliferator response element (PPRE) and upregulates gene transcription. Pioglitazone, an agonist of PPARG have recently suggested to improve locomotor function in a TDP-43 fly model [60].

Interestingly, dual treatment of Aliretinoin and Pranlukast may exert some additional protein regulation beyond those related with inflammation and excitotoxicity. This CD1 could play a role on regulation of ataxin-2 (ATX2). There is increasing evidence that alterations in RNA processing, predominantly mediated by the two RNA-binding proteins TDP-43 and FUS, may be relevant to ALS-frontotemporal dementia complex (FTLD) [61]. A potent modifier of the FUS pathology is an intermediate repeat length in the ATX2 gene. The survival motor neuron (SMN) protein may be also upregulated by CD1 by an unknown mechanism to be explored. SMN protein is significantly reduced in the spinal cord of patients with sporadic ALS, and neuronal overexpression of SMN significantly preserved locomotor function, rescued MNs, and attenuated astrogliosis in spinal cords of SOD1^G93A^ mice [62]. Another protein, VAPB, whose mutations have been linked to a loss of function in ALS, might be activated by CD1. ALS-linked mutated VAPB behaves in a dominant negative manner, sequestering wild type protein into cytoplasmic inclusions, and it was recently published a genetic network that includes other ALS genes such as *sod1*, *tdp43*, in addition to *tor* which is also a key regulator in autophagy and cell metabolism [63].

Recent successful results in the network biology field, such as the one addressed here, have attracted interest towards how systemic approaches might increase the revenue of the drug discovery process [14]. We found that *in silico* predicted combinations formed by Alitretinoin and Pranlukast (CD1) or Alitretinoin and Mefloquine (CD2) exerted effective neuroprotection in an *in vitro* model of ALS by reducing the toxic effects of chronic excitotoxicity on both microglia reactivity and MN survival. This drug repurposing seems of interest for further preclinical investigations with a perspective on clinical trials in patients.

## Conclusions

Here we demonstrated that systems biology approach is useful to find new drugs for the treatment of ALS. The polypharmacology proposed herein represented the first system-based targeting approach for ALS, which has not been addressed before. We demonstrated that *in silico* predictions are powerful enough to discover new and efficient therapies like the combinations proposed: Aliretinoin and Pranlukast or Aliretinoin and Mefloquine, which reduce microgliosis and rescue MNs under excitotoxic damage.

## Supporting Information

S1 Table Molecular characterization of the Pathophysiological motives causing ALS and effector proteins associated to each motive according to the literature search. (DOCX)

S2 Table Drug combinations for the treatment of ALS identified by TPMS. (DOCX)

S3 Table Synergy targets of the Alitretinoin Pranlukast (CD1). (DOCX)

S4 Table Synergy targets of the Alitretinoin and Mefloquine (CD2). (DOCX)

S5 Table Summary of binary drugs combination screening. (DOCX)
