# Supplementary material for: Novel Neuroprotective Multicomponent Therapy for Amyotrophic Lateral Sclerosis Designed by Networked Systems
Source: PLoS One. 2016 Jan 25;11(1):e0147626. doi: 10.1371/journal.pone.0147626 (PMC4726541; doi:10.1371/journal.pone.0147626)
Supplement: S1 Table — (DOCX) [file pone.0147626.s001.docx]

| **S1 Table. Molecular characterization of the Pathophysiological motives causing ALS and effector proteins associated to each motive according to the literature search** | | | | |
| --- | --- | --- | --- | --- |
| ALS pathophysiological motives | Nº of seeds | Short name | Uniprot ID | Reference |
| Glutamate excitotoxicity | 11 | **SLC1A2** | P43004 | [PMID: 16806844] |
|  |  | **GRIA1** | P42261 | [PMID: 16806844] |
|  |  | **GRIA2** | P42262 | [PMID: 16806844] |
|  |  | **GRIA3** | P42263 | [PMID: 16806844] |
|  |  | **GRIA4** | P48058 | [PMID: 16806844] |
|  |  | **SOD1** | P00441 | [PMID: 16806844] |
|  |  | **GRIN1** | Q05586 | [PMID: 16806844] |
|  |  | **GRIN2A** | Q12879 | [PMID: 16806844] |
|  |  | **GRIN2B** | Q13224 | [PMID: 16806844] |
|  |  | **GRIN2C** | Q14957 | [PMID: 16806844] |
|  |  | **GRIN2D** | O15399 | [PMID: 16806844] |
| Protein misfolding and aggregation | 23 | **SOD1** | P00441 | [PMID: 21296405] |
|  |  | **RNF19A** | Q9NV58 | [PMID:15217349] |
|  |  | **DERL1** | Q9BUN8 | [PMID: 18633404] |
|  |  | **MAP3K5** | Q99683 | [PMID: 18633404] |
|  |  | **TARDBP** | Q13148 | [PMID: 20864052] |
|  |  | **VCP** | P55072 | [PMID: 21144996] |
|  |  | **CSE1L** | P55060 | [PMID: 21700347] |
|  |  | **GRN** | P28799 | [PMID: 20349096] |
|  |  | **KPNA2** | P52292 | [PMID: 21700347] |
|  |  | **FUS** | P35637 | [PMID: 20864052] |
|  |  | **TNPO1** | Q92973 | [PMID: 21700347] |
|  |  | **OPTN** | Q96CV9 | [PMID: 20428114] |
|  |  | **NEFH** | P12036 | [OMIM: 162230] |
|  |  | **PRPH** | P41219 | [PMID: 20349096] |
|  |  | **FIG4** | Q92562 | [PMID: 20560784] |
|  |  | **BICD2** | Q8TD16 | [PMID: 20560784] |
|  |  | **ALS2** | Q96Q42 | [PMID: 20560784] |
|  |  | **MTOR** | P42345 | [PMID: 20560784] |
|  |  | **SQSTM1** | Q13501 | [PMID: 20560784] |
|  |  | **VAPB** | O95292 | [PMID: 20560784] |
|  |  | **CHMP2B** | Q9UQN3 | [PMID: 20560784][PMID: 20352044] |
|  |  | **HSPA1A** | P0DMV8 | [PMID:9930742] [PMID:18673445] |
|  |  | **HSPA1B** | P0DMV9 | [PMID:9930742] [PMID:18673445] |
| Mitochondrial dysfunction | 17 | **SOD1** | P00441 | [PMID: 20463400] |
|  |  | **KIF5B** | P33176 | [PMID: 20463400] |
|  |  | **MAPK11** | Q15759 | [PMID: 20463400] |
|  |  | **MAPK12** | P53778 | [PMID: 20463400] |
|  |  | **MAPK13** | O15264 | [PMID: 20463400] |
|  |  | **MAPK14** | Q16539 | [PMID: 20463400] |
|  |  | **DCTN1** | Q14203 | [PMID: 20463400] |
|  |  | **KIFAP3** | Q92845 | [PMID: 20463400] |
|  |  | **BCL2** | P10415 | [PMID: 20460269] |
|  |  | **Bcl-xL** | Q07817 | [PMID:20493207] |
|  |  | **XIAP** | P98170 | [PMID:20493207] |
|  |  | **Bad** | Q92934 | [PMID:20493207] |
|  |  | **Bax** | Q07812 | [PMID:20493207] |
|  |  | **cytochrome c** | P08574 | [PMID:20493207] |
|  |  | **caspase-3** | P42574 | [PMID:20493207] |
|  |  | **caspase-9** | P55211 | [PMID:20493207] |
|  |  | **ANG** | P03950 | [PMID: 21678416] |
| Oxidative Stress | 6 | **SOD1** | P00441 | [PMID: 18633404] |
|  |  | **RAC1** | P63000 | [PMID: 18633404] |
|  |  | **NOX1** | Q9Y5S8 | [PMID: 18633404] |
|  |  | **ERO1L** | Q96HE7 | [PMID: 20560784] |
|  |  | **P4HB** | P07237 | [PMID: 20560784] |
|  |  | **PDIA3** | P30101 | [PMID: 20560784] |
| Defective RNA processing | 9 | **ANG** | P03950 | [PMID: 20349096] |
|  |  | **ELP3** | Q9H9T3 | [PMID: 20349096] |
|  |  | **FUS** | P35637 | [PMID: 19840884] |
|  |  | **SETX** | Q7Z333 | [PMID: 20349096] |
|  |  | **SMN1** | Q16637 | [PMID: 20349096] |
|  |  | **TARDBP** | Q13148 | [PMID: 20349096] |
|  |  | **RGNEF** | Q8N1W1 | [PMID: 20349096] |
|  |  | **ATXN2** | Q99700 | [PMID: 20740002] |
|  |  | **C9orf72** | Q96LT7 | [PMID: 23181008] [PMID: 21944778] |
| Glial activation | 15 | **SLC1A2** | P43004 | [PMID: 19669429] |
|  |  | **SOD1** | P00441 | [PMID: 19669429] |
|  |  | **VEGFA** | P15692 | [PMID: 19669429]  [PMID:15351965] |
|  |  | **VEGFB** | P49765 | PMID: 19669429 PMID:18923022 |
|  |  | **TNF** | P01375 | [PMID: 19731042] |
|  |  | **IL1B** | P01584 | [PMID: 19731042] |
|  |  | **CHGA** | P10645 | [PMID: 19731042][PMID: 20807312] [PMID: 8721831] |
|  |  | **CHGB** | P05060 | [PMID: 20807312][PMID: 8721831] |
|  |  | **SCG2** | P13521 | [PMID: 20807312][PMID: 8721831] |
|  |  | **CD14** | P08571 | [PMID: 19731042] |
|  |  | **TLR2** | O60603 | [PMID: 19731042] |
|  |  | **IL4** | P05112 | [PMID: 19731042] |
|  |  | **IGF1** | P05019 | [PMID: 19731042] |
|  |  | **CX3CR1** | P49238 | [PMID: 19731042] |
|  |  | **CX3CL1** | P78423 | [PMID: 19731042] |
| Total number of seeds | 81 |  |  |  |
| Unique seeds | 72 |  |  |  |
