# Supplementary material for: Novel Neuroprotective Multicomponent Therapy for Amyotrophic Lateral Sclerosis Designed by Networked Systems
Source: PLoS One. 2016 Jan 25;11(1):e0147626. doi: 10.1371/journal.pone.0147626 (PMC4726541; doi:10.1371/journal.pone.0147626)
Supplement: S2 Table — (DOCX) [file pone.0147626.s002.docx]

| **S2 Table. Drug combinations for the treatment of ALS identified by TPMS** | | | | | | |
| --- | --- | --- | --- | --- | --- | --- |
|  | **Doses (µM)** | | **Vehicle** | | **Availability** | |
| **Code** | **Drug A** | **Drug B** | **DMSO** | **Cloroform** | **Drug A** | **Drug B** |
| CD1 | 1 | 1 | 0.005% | 0.006% | R4643 - Sigma | P0080 - Sigma |
| CD2 | 0.25 | 1 | 0.004% | --- | M2319 - Sigma | R4643 - Sigma |
