# Supplementary material for: Novel Neuroprotective Multicomponent Therapy for Amyotrophic Lateral Sclerosis Designed by Networked Systems
Source: PLoS One. 2016 Jan 25;11(1):e0147626. doi: 10.1371/journal.pone.0147626 (PMC4726541; doi:10.1371/journal.pone.0147626)
Supplement: S3 Table — (DOCX) [file pone.0147626.s003.docx]

| **S3 Table. Synergy targets of the Alitretinoin + Pranlukast (CD1)** | | |
| --- | --- | --- |
| **UniProt** | **Gene name** | **Score** |
| **O43318** | M3K7 | 0,328947 |
| **O95292** | VAPB | 0,328947 |
| **P45983** | MK08 | 0,328947 |
| **P45984** | MK09 | 0,328947 |
| **P53779** | MK10 | 0,328947 |
| **Q15750** | TAB1 | 0,328947 |
| **Q8N5C8** | TAB3 | 0,328947 |
| **P01584** | IL1B | 0,282895 |
| **P08574** | CY1 | 0,282895 |
| **P15692** | VEGFA | 0,282895 |
| **Q04206** | TF65 | 0,282895 |
| **Q09472** | EP300 | 0,282895 |
| **Q16637** | SMN | 0,282895 |
| **Q9NYJ8** | TAB2 | 0,282895 |
| **Q99683** | M3K5 | 0,282895 |
| **Q99700** | ATX2 | 0,282895 |
| **Q07817** | B2CL1 | 0,282895 |
| **O15392** | BIRC5 | 0,281955 |
| **Q9NV58** | RN19A | 0,281955 |
| **Q92845** | KIFA3 | 0,281955 |
| **P00533** | EGFR | 0,236842 |
| **P04150** | GCR | 0,236842 |
| **P08138** | TNR16 | 0,236842 |
| **P30153** | 2AAA | 0,236842 |
| **P30154** | 2AAB | 0,236842 |
| **P42224** | STAT1 | 0,236842 |
| **P62714** | PP2AB | 0,236842 |
| **P63151** | 2ABA | 0,236842 |
| **P67775** | PP2AA | 0,236842 |
| **Q00005** | 2ABB | 0,236842 |
| **Q14790** | CASP8 | 0,236842 |
| **Q16539** | MK14 | 0,236842 |
| **Q66LE6** | 2ABD | 0,236842 |
| **Q9Y2T4** | 2ABG | 0,236842 |
| **P55072** | TERA | 0,235902 |
| **Q14203** | DCTN1 | 0,235902 |
| **Q96CV9** | OPTN | 0,235902 |
| **P40763** | STAT3 | 0,234962 |
| **Q13490** | BIRC2 | 0,192669 |
| **Q07812** | BAX | 0,191729 |
| **O15264** | MK13 | 0,190789 |
| **P01116** | RASK | 0,190789 |
| **P13500** | CCL2 | 0,190789 |
| **P14780** | MMP9 | 0,190789 |
| **P53778** | MK12 | 0,190789 |
| **P63000** | RAC1 | 0,190789 |
| **Q05397** | FAK1 | 0,190789 |
| **Q15759** | MK11 | 0,190789 |
| **Q92583** | CCL17 | 0,190789 |
| **P28482** | MK01 | 0,18891 |
| **P41219** | PERI | 0,18891 |
| **Q92562** | FIG4 | 0,18891 |
| **P19438** | TNR1A | 0,145677 |
| **P04637** | P53 | 0,144737 |
| **P04839** | CY24B | 0,144737 |
| **P04899** | GNAI2 | 0,144737 |
| **P05412** | JUN | 0,144737 |
| **P13498** | CY24A | 0,144737 |
| **P13501** | CCL5 | 0,144737 |
| **P17252** | KPCA | 0,144737 |
| **P19320** | VCAM1 | 0,144737 |
| **P19878** | NCF2 | 0,144737 |
| **P35354** | PGH2 | 0,144737 |
| **P42574** | CASP3 | 0,144737 |
| **P62873** | GBB1 | 0,144737 |
| **Q05586** | NMDZ1 | 0,144737 |
| **Q12879** | NMDE1 | 0,144737 |
| **Q15080** | NCF4 | 0,144737 |
| **Q9HBY0** | NOX3 | 0,144737 |
| **Q9Y5S8** | NOX1 | 0,144737 |
| **P35222** | CTNB1 | 0,144737 |
| **P29353** | SHC1 | 0,143797 |
| **P52292** | IMA1 | 0,143797 |
| **P07237** | PDIA1 | 0,142857 |
| **P00441** | SODC | 0,141917 |
| **P14778** | IL1R1 | 0,141917 |
| **P27930** | IL1R2 | 0,141917 |
| **Q9UL17** | TBX21 | 0,141917 |
| **P49765** | VEGFB | 0,141917 |
| **Q96HE7** | ERO1A | 0,140977 |
| **P0CG47** | UBB | 0,098684 |
| **P01375** | TNFA | 0,098684 |
| **P08107** | HSP71 | 0,098684 |
| **P10415** | BCL2 | 0,098684 |
| **P16520** | GBB3 | 0,098684 |
| **P31749** | AKT1 | 0,098684 |
| **P31751** | AKT2 | 0,098684 |
| **Q12933** | TRAF2 | 0,098684 |
| **Q14957** | NMDE3 | 0,098684 |
| **Q9Y4K3** | TRAF6 | 0,098684 |
| **P01112** | RASH | 0,097744 |
| **O15399** | NMDE4 | 0,097744 |
| **P01111** | RASN | 0,097744 |
| **Q7Z333** | SETX | 0,097744 |
| **Q16665** | HIF1A | 0,095865 |
| **P01583** | IL1A | 0,095865 |
| **P02778** | CXL10 | 0,095865 |
| **Q07325** | CXCL9 | 0,095865 |
| **Q9H9T3** | ELP3 | 0,095865 |
| **O14727** | APAF | 0,094925 |
| **P00519** | ABL1 | 0,094925 |
| **P17612** | KAPCA | 0,094925 |
| **P33176** | KINH | 0,094925 |
| **P98170** | XIAP | 0,094925 |
| **Q00653** | NFKB2 | 0,094925 |
| **Q92973** | TNPO1 | 0,094925 |
| **P30101** | PDIA3 | 0,093985 |
| **P60953** | CDC42 | 0,093985 |
| **Q04637** | IF4G1 | 0,093985 |
| **P01138** | NGF | 0,093985 |
| **O00329** | PK3CD | 0,052632 |
| **O00459** | P85B | 0,052632 |
| **O14610** | GBGT2 | 0,052632 |
| **O14775** | GBB5 | 0,052632 |
| **O60262** | GBG7 | 0,052632 |
| **P04049** | RAF1 | 0,052632 |
| **P08754** | GNAI3 | 0,052632 |
| **P09471** | GNAO | 0,052632 |
| **P14598** | NCF1 | 0,052632 |
| **P19838** | NFKB1 | 0,052632 |
| **P27986** | P85A | 0,052632 |
| **P42336** | PK3CA | 0,052632 |
| **P42338** | PK3CB | 0,052632 |
| **P48736** | PK3CG | 0,052632 |
| **P50150** | GBG4 | 0,052632 |
| **P50151** | GBG10 | 0,052632 |
| **P59768** | GBG2 | 0,052632 |
| **P61586** | RHOA | 0,052632 |
| **P61952** | GBG11 | 0,052632 |
| **P62879** | GBB2 | 0,052632 |
| **P63211** | GBG1 | 0,052632 |
| **P63215** | GBG3 | 0,052632 |
| **P63218** | GBG5 | 0,052632 |
| **Q02750** | MP2K1 | 0,052632 |
| **Q13233** | M3K1 | 0,052632 |
| **Q8WYR1** | PI3R5 | 0,052632 |
| **Q9HAV0** | GBB4 | 0,052632 |
| **Q9P2W3** | GBG13 | 0,052632 |
| **Q9UBI6** | GBG12 | 0,052632 |
| **Q9UK08** | GBG8 | 0,052632 |
| **Q92569** | P55G | 0,052632 |
| **Q92934** | BAD | 0,052632 |
| **P10398** | ARAF | 0,051692 |
| **Q00987** | MDM2 | 0,051692 |
| **Q12967** | GNDS | 0,051692 |
| **Q13224** | NMDE2 | 0,051692 |
| **Q15052** | ARHG6 | 0,051692 |
| **P49238** | CX3C1 | 0,051692 |
| **P62993** | GRB2 | 0,050752 |
| **P05019** | IGF1 | 0,050752 |
| **P06748** | NPM | 0,050752 |
| **Q9UER7** | DAXX | 0,050752 |
| **P27361** | MK03 | 0,049812 |
| **Q92831** | KAT2B | 0,048872 |
| **Q99836** | MYD88 | 0,048872 |
| **P09341** | GROA | 0,047932 |
| **P15923** | TFE2 | 0,047932 |
| **P22736** | NR4A1 | 0,047932 |
| **O15379** | HDAC3 | 0,046992 |
| **O95155** | UBE4B | 0,046992 |
| **O95163** | ELP1 | 0,046992 |
| **P04114** | APOB | 0,046992 |
| **P05231** | IL6 | 0,046992 |
| **P08253** | MMP2 | 0,046992 |
| **P10147** | CCL3 | 0,046992 |
| **P12318** | FCG2A | 0,046992 |
| **P12931** | SRC | 0,046992 |
| **P13747** | HLAE | 0,046992 |
| **P16298** | PP2BB | 0,046992 |
| **P30484** | 1B46 | 0,046992 |
| **P30505** | 1C08 | 0,046992 |
| **P49407** | ARRB1 | 0,046992 |
| **P49840** | GSK3A | 0,046992 |
| **Q14289** | FAK2 | 0,046992 |
| **Q29836** | 1B67 | 0,046992 |
| **Q8NEZ2** | VP37A | 0,046992 |
| **Q9GZP9** | DERL2 | 0,046992 |
| **Q9UQN3** | CHM2B | 0,046992 |
| **Q92556** | ELMO1 | 0,046992 |
| **Q92608** | DOCK2 | 0,046992 |
| **Q92985** | IRF7 | 0,046992 |
