# Supplementary material for: Novel Neuroprotective Multicomponent Therapy for Amyotrophic Lateral Sclerosis Designed by Networked Systems
Source: PLoS One. 2016 Jan 25;11(1):e0147626. doi: 10.1371/journal.pone.0147626 (PMC4726541; doi:10.1371/journal.pone.0147626)
Supplement: S4 Table — (DOCX) [file pone.0147626.s004.docx]

| **S4 Table. Synergy targets of the Alitretinoin and Mefloquine (CD2)** | | |
| --- | --- | --- |
| **UniProt** | **Gene name** | **Score** |
| **P45983** | MK08 | 0,275219 |
| **P45984** | MK09 | 0,275219 |
| **P53779** | MK10 | 0,275219 |
| **Q16539** | MK14 | 0,248355 |
| **Q15759** | MK11 | 0,239401 |
| **P05412** | JUN | 0,239035 |
| **P53778** | MK12 | 0,230446 |
| **O15264** | MK13 | 0,230446 |
| **P63000** | RAC1 | 0,229532 |
| **P14780** | MMP9 | 0,201754 |
| **P04049** | RAF1 | 0,168494 |
| **Q04206** | TF65 | 0,167763 |
| **O00459** | P85B | 0,158808 |
| **P42336** | PK3CA | 0,158808 |
| **Q99683** | M3K5 | 0,149854 |
| **O00329** | PK3CD | 0,149854 |
| **P48736** | PK3CG | 0,149854 |
| **P42338** | PK3CB | 0,149854 |
| **Q92569** | P55G | 0,149854 |
| **P42261** | GRIA1 | 0,138889 |
| **P48058** | GRIA4 | 0,138341 |
| **P42574** | CASP3 | 0,132127 |
| **Q9Y4K3** | TRAF6 | 0,131944 |
| **Q9Y5S8** | NOX1 | 0,131396 |
| **Q12879** | NMDE1 | 0,129934 |
| **Q9UKW4** | VAV3 | 0,127924 |
| **P52735** | VAV2 | 0,127924 |
| **P19838** | NFKB1 | 0,12299 |
| **P19878** | NCF2 | 0,122442 |
| **P15498** | VAV | 0,118969 |
| **P13498** | CY24A | 0,113487 |
| **P04839** | CY24B | 0,113487 |
| **P14598** | NCF1 | 0,104532 |
| **P28482** | MK01 | 0,103253 |
| **P40763** | STAT3 | 0,102705 |
| **P04637** | P53 | 0,096308 |
| **Q92793** | CBP | 0,086988 |
| **P01116** | RASK | 0,086075 |
| **P00533** | EGFR | 0,084064 |
| **P01584** | IL1B | 0,077303 |
| **Q14957** | NMDE3 | 0,076389 |
| **P29353** | SHC1 | 0,075841 |
| **Q05586** | NMDZ1 | 0,074561 |
| **P01112** | RASH | 0,069079 |
| **P01111** | RASN | 0,068165 |
| **P01375** | TNFA | 0,066155 |
| **Q07817** | B2CL1 | 0,060307 |
| **P10415** | BCL2 | 0,060124 |
| **P15056** | BRAF | 0,059942 |
| **P17252** | KPCA | 0,058845 |
| **P0CG47** | UBB | 0,058662 |
| **Q13224** | NMDE2 | 0,057931 |
| **Q16665** | HIF1A | 0,057749 |
| **Q09472** | EP300 | 0,056652 |
| **Q9UBK2** | PRGC1 | 0,048246 |
| **P60953** | CDC42 | 0,046235 |
| **P02647** | APOA1 | 0,045687 |
| **Q92934** | BAD | 0,041484 |
| **P31749** | AKT1 | 0,040936 |
| **P35222** | CTNB1 | 0,040753 |
| **Q05397** | FAK1 | 0,040753 |
| **P19174** | PLCG1 | 0,038743 |
| **Q14203** | DCTN1 | 0,03856 |
| **P27361** | MK03 | 0,037646 |
| **P04150** | GCR | 0,037463 |
| **P08107** | HSP71 | 0,033443 |
| **P42224** | STAT1 | 0,032529 |
| **P04629** | NTRK1 | 0,029971 |
| **P61586** | RHOA | 0,02924 |
| **P01100** | FOS | 0,028143 |
| **Q96CV9** | OPTN | 0,027961 |
| **P08574** | CY1 | 0,027778 |
| **O95292** | VAPB | 0,027778 |
| **Q92562** | FIG4 | 0,027778 |
| **P41219** | PERI | 0,027595 |
| **Q9H9T3** | ELP3 | 0,027595 |
| **Q96HE7** | ERO1A | 0,027595 |
| **P16220** | CREB1 | 0,023575 |
| **P09471** | GNAO | 0,022478 |
| **Q9HAV0** | GBB4 | 0,022478 |
| **P50151** | GBG10 | 0,022478 |
| **P50150** | GBG4 | 0,022295 |
| **Q9UBI6** | GBG12 | 0,022295 |
| **O14775** | GBB5 | 0,022295 |
| **O60262** | GBG7 | 0,022295 |
| **O14610** | GBGT2 | 0,022295 |
| **P59768** | GBG2 | 0,022295 |
| **P62879** | GBB2 | 0,022295 |
| **P63215** | GBG3 | 0,022295 |
| **P61952** | GBG11 | 0,022295 |
| **P63096** | GNAI1 | 0,022295 |
| **Q9UK08** | GBG8 | 0,022295 |
| **P63218** | GBG5 | 0,022295 |
| **P63211** | GBG1 | 0,022295 |
