# Supplementary material for: Novel Neuroprotective Multicomponent Therapy for Amyotrophic Lateral Sclerosis Designed by Networked Systems
Source: PLoS One. 2016 Jan 25;11(1):e0147626. doi: 10.1371/journal.pone.0147626 (PMC4726541; doi:10.1371/journal.pone.0147626)
Supplement: S5 Table — (DOCX) [file pone.0147626.s005.docx]

| S5 Table. Summary of binary drugs combination screening. | | | |
| --- | --- | --- | --- |
| Category | Interactome analysis | | |
|  | ALS | Glutamate excitotxicity | RNA processing |
| Starting number of binary combinations | 14,794,080 | 14,794,080 | 14,794,080 |
| Binary combinations with combinations that contain non-synonymous drugs with the following characteristics:   - Drugs with an outstanding safety profile - Drugs with at least one known human target - Drugs with known interactions - Drugs with the known therapeutic target inside the human biological network. | 134,940 | 134,940 | 134,940 |
| Binary combinations of approved drugs with a relationship with ALS, and with a supra-additive effect between the two drugs of the combination | 593 | 42 | 7 |
| Binary combinations composed by approved drugs that cross the blood brain barrier or unknown information regarding BBB permeability. | 447 | 39 | 6 |
| Binary combinations not previously related to ALS with a plausible MoA for treating ALS, or the corresponding ALS motive | 7 | 4 | 1 |
| Total number of binary combinations | 12 | | |
